# Comparative analysis of gender disparity in academic positions based on U.S. region and STEM discipline

**DOI:** 10.1371/journal.pone.0298736

**Published:** 2024-03-20

**Authors:** Danielle J. Galvin, Susan C. Anderson, Chelsi J. Marolf, Nikole G. Schneider, Andrea L. Liebl

**Affiliations:** Department of Biology, University of South Dakota, Vermillion, South Dakota, United States of America; Instituto Tecnologico Autonomo de Mexico, MEXICO

## Abstract

Despite a move toward gender parity in the United States (U.S.) workforce, a large gender gap persists in the fields of science, technology, engineering, and mathematics (STEM); this is particularly true for academic (i.e., instructor and tenure track) STEM positions. This gap increases as women advance through the traditional steps of academia, with the highest degree of gender disparity in tenured positions. As policies, politics, and culture, which all contribute to gender equity across the world, vary across regions in the United States, we expect that the gender gap in STEM might also vary across geographic regions. Here, we evaluated over 20,000 instructor and tenure track positions in university STEM departments across the U.S. to evaluate whether and how the geographic region of a university might determine its proportion of women in STEM academic positions. Similar to previous research, regardless of geographic region, more men were employed in both tenure track and instructor positions across STEM fields. However, variation existed regionally within the U.S., with the Mountain region employing the lowest proportion of women in tenure track positions and the East North Central and Pacific regions employing the greatest proportion. We expect this regional variation could be caused by differences in state and local policies, regional representation, and mentorship, resulting in inconsistent support for women, leading to differences in work environments, hiring, and job retention rates across the country. A better understanding of which geographic areas within the U.S. have more equal distributions of women in the STEM field will help us to identify the specific mechanisms that facilitate more equal and inclusive opportunities for women and other underrepresented groups across all levels of STEM academia.

## Introduction

Gender inequality is a persistent problem throughout the United States workforce, particularly in the STEM (Science, Technology, Engineering, and Mathematics) fields, where large gender gaps are especially evident in STEM academic positions [1–3]. Despite women making up about 50% of assistant professors in STEM fields, that number decreases in tenured positions to 45.4% of associate and 32.8% of full STEM professors [4]. Identifying the underlying cause(s) for disparities in STEM academia is particularly important both for the women themselves and for the quality of the research they produce. Scientists from different backgrounds contribute more diverse research perspectives that can reduce biases that have been shown to exist in scientific research. For example, birdsong was traditionally thought to be an exclusively male trait, but recent studies (primarily by women authors) show that, in many species, female birds are also capable of song [4], completely revolutionizing the field. To increase gender equality in academic STEM fields, generally, we must first understand where the discrepancies exist and the potential causes, be it social and cultural norms surrounding women’s and men’s roles, policies and laws influencing hiring processes in academia, or other obstacles that prevent women from advancing their STEM careers. As many of these factors are expected to vary culturally, and therefore with geographic region, our goal in this study was to identify whether the proportion of women employed in STEM academia varied by geographic region across the United States (U.S.); using this information, future research might begin to identify some of the specific factors that might contribute to differences observed.

The term commonly used to describe the loss of women in STEM academia is the “leaky pipeline,” which refers to the attrition of women as they move through the “traditional” steps of academia (i.e., undergraduate, graduate, post-doctorate, into tenure track positions) [5–7]. However, not all steps have the same level of attrition [8] and multiple, interacting factors contribute to this attrition. For instance, hiring practices for STEM tenure track jobs can be biased against women with the use of masculine wording in job advertisements [9, 10] and biases in the evaluation of job candidates; even when candidates’ qualifications are identical, male candidates are selected for interviews and job offers more often by hiring committees [1, 11]. When offered jobs, women are offered lower start-up packages [9, 10] and lower salaries than their male counterparts [12]. Job offers (including start-up packages and salary) are thought to be lower as women are less likely to negotiate [13, 14], particularly when they are negotiating with men [15]. Additionally, women scientists are more likely to be married to another scientist [13], leading to the “two body problem” which makes jobs even more difficult to obtain. Once in tenure track academic jobs, women are expected to take on higher mentoring and service loads than their male colleagues despite no compensation, potentially because women are thought to be ‘caretakers’ whereas men are seen mores as ‘task-masters’ [15]. This increase in service load can cause an increase in burnout, resulting in women leaving academia at a higher rate. Finally, the culture of academia (e.g., how colleagues address each other, collaboration, comradery) has been considered a factor in pushing women to leave science altogether [3, 16–18]. As a result, women tend to have less job satisfaction and higher turnover [19]. These factors likely vary institutionally, and possibly geographically, in relation to regional policies, culture, and politics [20], resulting in increased gender equity in areas where more programs supporting women are offered. Conversely, regions with generally more distrust for science may have more gender imbalance as a result of societal gender norms and culture within scientific disciplines [20].

In addition to challenges associated with getting and maintaining academic jobs, family obligations are a greater burden for women than men. Women applying for academic positions who have children or other dependents can experience impediments during the recruitment process, whereas men with children are less affected [7, 21–23]. As a result, women are more likely to accept positions that provide better access to health care, family-leave, and childcare; such resources and benefits often vary among states [24, 25], which could lead to regional differences in the number of women in scientific careers. Finally, women are more willing to make career sacrifices for familial obligations than men, such as denying or leaving tenure positions for a more flexible instructor or part-time employment, resulting in an overrepresentation of women in instructor-level positions [13].

Although it is well-established that there are fewer women in STEM academic positions, we do not know if this disparity is uniform across the United States. Here, we predict that differences exist across regions of the U.S. that correspond with cultural or political differences in those regions, particularly as the country becomes more politically polarized. By first understanding how regional differences can lead to variation in parity of women in academic STEM fields, we might better address which policies and cultural traits reduce or exaggerate the effect of fewer women in the STEM fields. Using geographical region as defined by the U.S. census and university websites, we determined the proportion of women in tenure track and instructor positions in each region across STEM disciplines to assess if regionality could be a contributing factor for the gender disparity seen in academic employment and retention in STEM. We acknowledge that gender does not exist on a binary spectrum [26]and individuals who do not identify as either “male” or “female” may feel even more marginalized than women. However, here, we assigned gender based on pronouns, names, and photographs listed on departmental websites. Although this may not accurately represent an individual’s self-identified gender, our goal was to determine the perceived gender balance (or imbalance) of an academic department as it would be viewed [26, 27] by potential students, applicants, or collaborating institutions.

## Material and methods

### Gender ratios

In this study, we determined the number of men and women faculty in STEM departments at 127 universities. We assigned gender using published photos, names, and listed pronouns of faculty in STEM departments on university websites, rather than using self-identifying methods (e.g., through surveys). We recognize that the way in which we use the term “gender” significantly impacts the context of this study [21, 27, 28]. Here, we define gender as societally perceived assumptions of sex differences, as outlined in the current addition of the Publication Manual of the American Psychological Association (APA) [29] and in agreement with several foundational works which argue that gender describes a nonphysiological, categorical, culturally constructed term by which we distinguish groups of people [26, 30]. We acknowledge that although gender should not be considered strictly binary, assumptions were made regarding the gender identity of the individuals in this study by necessity to create a robust sample size, and we argue that those using a departmental website to gain information about that department (e.g., prospective students and colleagues applying to a department) would do the same [27]. As the number of women in a field impacts both trainees and potential co-workers [9, 31], this information is valuable. Of the 21,512 individuals for whom we determined gender, we could only verify a single individual who openly identified with “they/them” pronouns, who was excluded from analysis.

We identified gender for both instructors and tenure track faculty in STEM departments (biology, chemistry, computer science, engineering, mathematics, and physics) at universities across each of the nine geographic regions designated by the U.S. Census Bureau [32]. For each university (S1 Table), we visited each STEM department’s website between March and November of 2022. We then counted the number of men and women in instructor level and tenure track positions in each department. We assigned gender using photographs and listed names on university websites; when a photograph was unavailable, we used personal websites and/or student reviews from ratemyprofessor.com to determine gender.

In an effort to survey a representative subsample of the thousands of higher education institutions across the United States, we used a stratified random sample design to choose universities (n = 127) which varied in size (number of students) and level of research activity across each geographic region (S1 Table). Universities were classified by size as small (<1000 students), medium (1000–9999 students), or large (≥10000 students) and by research activity level as primarily undergraduate institutions (PUI), high research activity institutions (R2), or very high research activity institutions (R1) as designated by the American Council on Education [33]. Ensuring a balanced distribution of university data collected from each region is important, because tenure requirements can vary based on research classification [33, 34]; for instance, PUIs often require higher teaching loads, more service, and more advising to achieve tenure, whereas R1 and R2 universities emphasize research productivity along with advising, service, and teaching [34]. We selected our representative sample to encompass universities among each classification in every region.

### Data analysis

Several institutions had cross-listed computer science and engineering departments, which included identical faculty members. In these cases, and when the department of computer science was classified as part of the College of Engineering, the data was combined and listed as a single department of engineering. However, if the department of computer science was clearly delineated from engineering (e.g., in a College of Arts and Sciences), it was designated as its own department and the data was listed separately for computer science and engineering. One institution had a cross-listed department of chemistry and physics, which was excluded from the analysis.

Data were analyzed using R (version 4.1.3 [35]) in RStudio (version 2022.07.1 [36]). All models were fit using *rstan* (version 2.21.3 [37]) with the *brms* package (version 2.16.3 [38]). To investigate how the proportion of women employed varied by region, STEM department, and position, we used a Bayesian generalized linear model (GLM) with a binomial likelihood and a logit link, due to the binary (male/female) nature of our data. We further analyzed this question by determining how the absolute number of women employed (rather than proportion) differed based on region, position, and STEM department using a Bayesian GLM with a Poisson likelihood and a logistic link. We used Bayesian methods because we were interested in estimating the probability of our hypotheses being true [39]. Priors for these models were selected using prior simulation [40]. Briefly, N values were drawn and for each value, a model outcome was simulated, and the results were plotted. This plot was assessed based on prior knowledge (i.e., that the population of the United States is approximately 50% female, indicating that the proportion of women employed in tenure track and instructor positions should be similar [41]) and a final determination made regarding how well the results represent prior knowledge. When the simulated values correctly reflected prior knowledge, the data was added to the model to estimate the posterior distribution [40]. Markov chain Monte Carlo (MCMC) simulation was used to obtain the joint posterior distribution (which quantifies uncertainty between the observed and simulated values and is used in calculating the values of interest [42]) from which all reported values were calculated. Rhat values were used to assess model convergence (with Rhat <1.01, indicating convergence). Values for the mean and 95% credible intervals were calculated using the posterior distribution [43]. The difference between the mean proportion of women employed in each STEM field for each region was calculated over 4000 iterations and 4 chains of posterior distribution. To calculate the probability of these values, the number of differences greater than zero was divided by the total number of samples in the distribution (n = 16000). All data are presented as proportions with the associated 95% credible interval.

## Results

**Fig 1:**
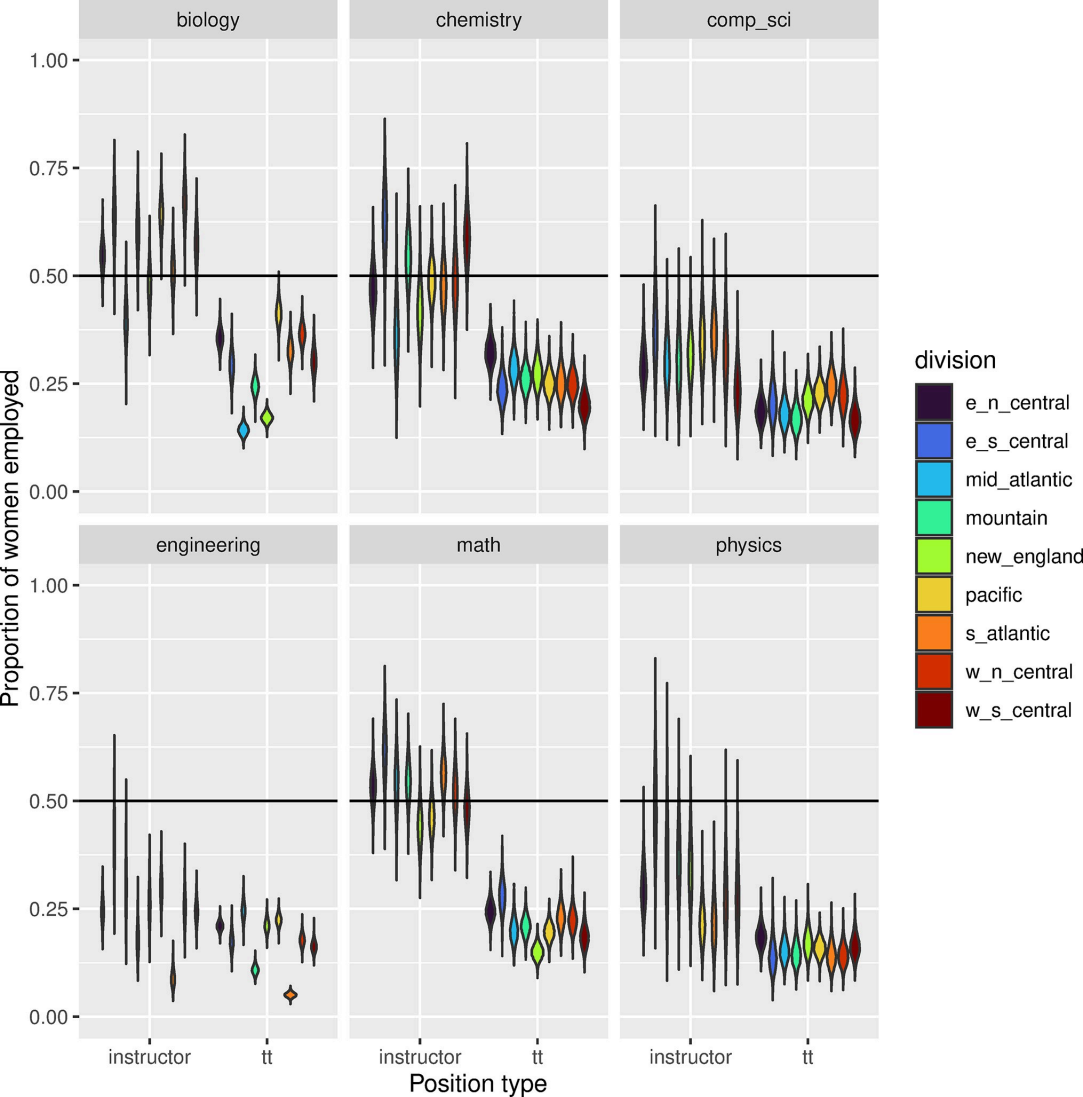
Violin plot showing the differences between the proportion of women employed in tenure track and instructor positions for each STEM department and division of the U.S. The horizontal line denotes a proportion of 0.5, the goal for gender parity.

The proportion of women employed varied by position, STEM field, and region of the U.S. (Fig 1). Unsurprisingly, there was a >99.9% probability that more men were employed in tenure track positions than women, regardless of STEM field or region of the U.S. (S2 Table). For instructor positions, there was a 62.9% probability that more men were employed in instructor positions than women, also regardless of STEM field or region of the U.S. (S2 Table).

**Fig 2:**
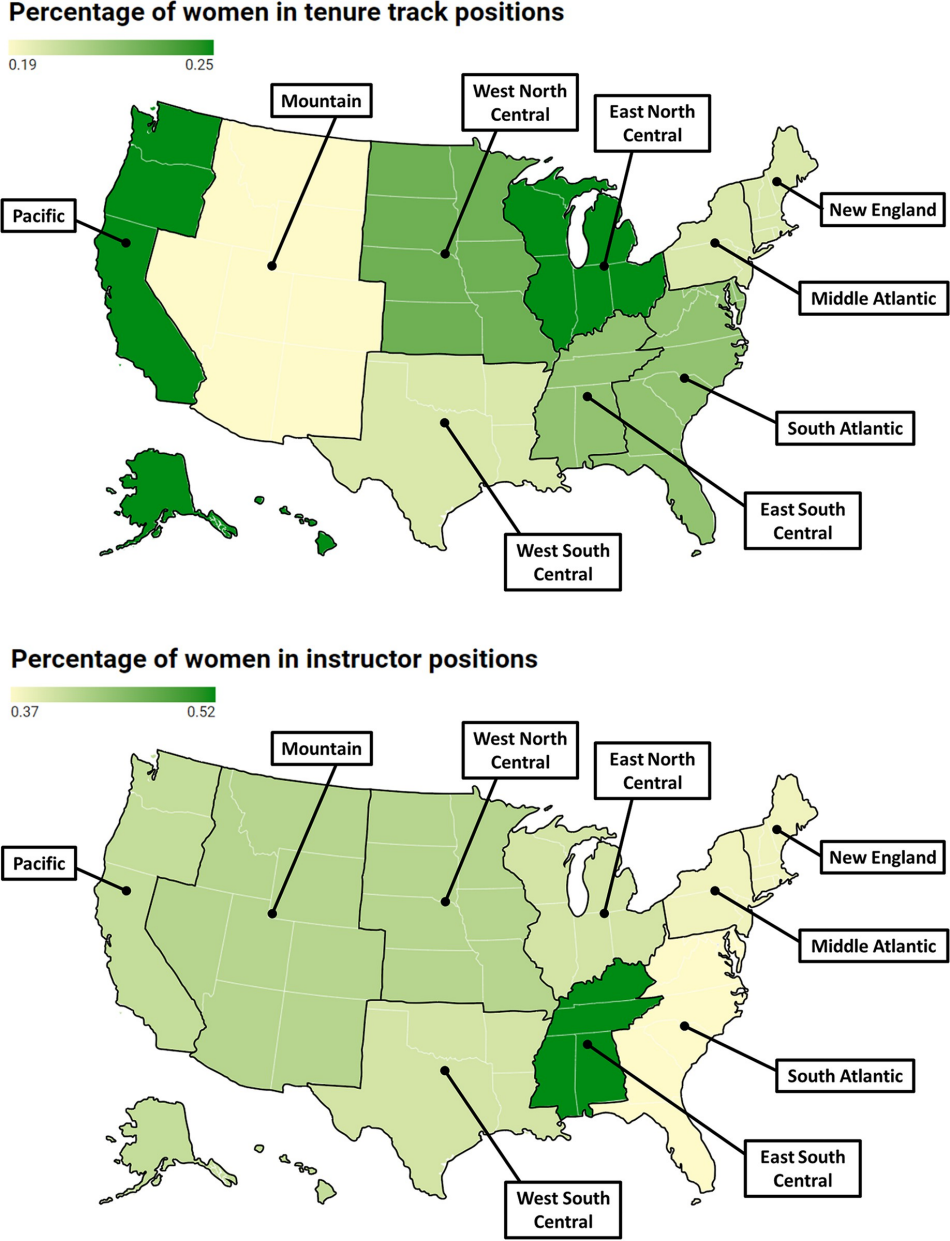
Heat map depicting the average proportion of women employed in (a) instructor and (b) tenure track positions based on region. Darker coloring indicates a greater proportion of women employed in that region than surrounding regions. Figures created with Datawrapper [44].

Regionally, we found differences in the proportion of women working in STEM departments across the country. In instructor positions across all STEM fields, the East South Central region (AL, KY, MS, TN) employed the greatest proportion of women (0.52, 95% CrI: 0.29 to 0.73), whereas the South Atlantic region (DC, DE, FL, GA, MD, NC, SC, VA, WV) employed the lowest (0.37, 95% CrI: 0.07 to 0.61, Fig 2A; Table 1). In tenure track positions, the East North Central (IL, IN, MI, OH, WI) and Pacific (AK, CA, HI, OR, WA) regions employed the greatest proportion of women (although this was only 0.25, 95% CrI: 0.15 to 0.38, 0.14 to 0.44, respectively) across all STEM fields, whereas the Mountain region (AZ, CO, ID, MT, NM, NV, UT, WY) employed the lowest proportion (0.19, 95% CrI: 0.10 to 0.29, Fig 2B; Table 1).

**Table 1:** Average proportion and the associated 95% credible interval of women employed in tenure track and instructor positions across all STEM disciplines by region.

Region	States	Tenure Track	Instructor
East North Central	Illinois, Indiana, Michigan, Ohio, and Wisconsin	0.25 (0.15–0.38)	0.40 (0.21–0.59)
East South Central	Alaska, Kentucky, Mississippi, and Tennessee	0.22 (0.11–0.33)	0.52 (0.29–0.73)
Middle Atlantic	New Jersey, New York, and Pennsylvania	0.20 (0.12–0.33)	0.38 (0.21–0.61)
Mountain	Arizona, Colorado, Idaho, Montana, New Mexico, Nevada, Utah, and Wyoming	0.19 (0.10–0.29)	0.42 (0.15–0.66)
New England	Connecticut, Massachusetts, Maine, New Hampshire, Rhode Island, and Vermont	0.20 (0.13–0.31)	0.38 (0.21–0.54)
Pacific	Alaska, California, Hawaii, Oregon, and Washington	0.25 (0.14–0.44)	0.41 (0.18–0.68)
South Atlantic	Delaware, Florida, Georgia, Maryland, North Carolina, South Carolina, Virginia, Washington D.C., and West Virginia	0.22 (0.05–0.35)	0.37 (0.07–0.61)
West North Central	Iowa, Kansas, Minnesota, Missouri, North Dakota, Nebraska, and South Dakota	0.23 (0.12–0.39)	0.42 (0.19–0.72)
West South Central	Arkansas, Louisiana, Oklahoma, and Texas	0.20 (0.13–0.33)	0.40 (0.17–0.65)

**Fig 3:**
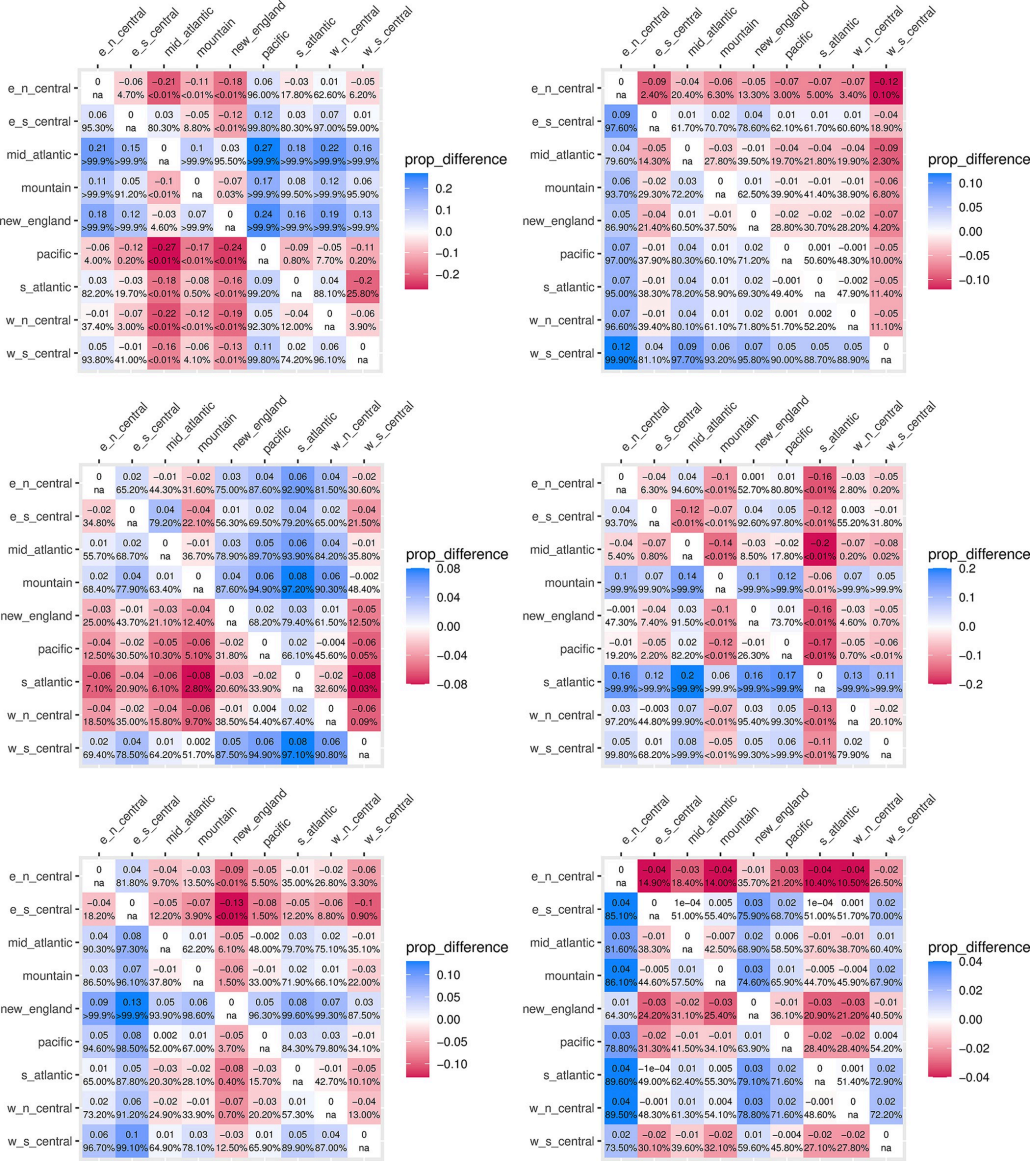
Heat maps showing differences in the proportion of women employed in tenure track positions across different regions in the STEM fields of biology (a), chemistry (b), computer science (c), engineering (d), mathematics (e) and physics (f). Darker tiles represent greater disparity in the proportion of women employed in tenure track positions with blue indicating the region on the x-axis employs more women and red indicating the region on the x-axis employs fewer. Hence, the values in blue tiles are always positive and red tiles negative. Each box includes the difference in proportion of women employed, followed by the probability of those differences (as a percent). The further this probability is from 50%, the stronger the likelihood of the difference between regions. For instance, comparing biology departments between the Pacific and Middle Atlantic regions, a notable difference is seen by the dark blue shading of their corresponding tile. A positive value of 0.27 indicates that 27% more women are employed in tenure track positions in the Pacific region compared to the Middle Atlantic region.

The distribution of women in either tenure track or instructor level positions across regions was not equal for all STEM fields (Fig 3). Two geographic areas consistently (in all or nearly all departments) employed a greater proportion of women compared to other areas: Pacific and East North Central. Although there were no STEM departments in any region that employed more women than men in tenure track positions, in some regions of the U.S., there was a higher proportion of women employed as instructors in biology, chemistry, and mathematics (Fig 1). Interestingly, in tenure track positions, biology had more variability in the proportion of women employed across geographic regions than any other discipline (0.14–0.41, Fig 3; S2 Table). Physics showed the least variation in the proportion of women employed in tenure track positions across regions (0.14–0.19, Fig 3; S2 Table) but had the lowest overall proportion of women employed in tenure track positions (Fig 1). In three out of the eight STEM fields (biology, chemistry, and physics), the East North Central region had a higher proportion of women in tenure track positions compared to all other regions (Fig 3), whereas the West South Central region (AR, LA, OK, TX) had a lower proportion of women employed in tenure track positions in computer science and chemistry compared to all other regions (Fig 3).

## Discussion

Although the disparity in the proportion of women in academic tenure track positions has been illustrated previously [2, 16, 45–47], we show here that this disparity is not uniform across the United States. The imbalance was most prevalent in computer science, engineering, and physics, where our data showed less than 25% of tenure track positions are held by women. Two geographic areas, Pacific (AK, CA, HI, OR, WA) and East North Central (IL, IN, MI, OH, WI), employed a greater proportion of women than other regions in all or nearly all departments, resulting in greater gender parity in tenure track positions in these two regions. However, the highest proportion of women in those regions were 0.41 and 0.36, respectively, indicating that even the areas of the country that are closest to gender parity in academic STEM fields are still not equal. In instructor positions, some regions showed equal or greater proportions of women, but this was isolated to the fields of biology, chemistry, and mathematics. Variability in the proportion of women employed across region, discipline, and position in our data was large, with 5% women in engineering tenure track positions in the South Atlantic to 67% women in biology instructor positions in the West North Central. These departmental differences are consistent with studies on every level of academia in STEM fields, from undergraduate participation through tenured faculty [17, 48–51]. Factors contributing to the regional differences are likely numerous and complex, but we have identified several policies, initiatives, and demographic variables that may play a role in which regions women in academia choose to work.

Despite progress in social and cultural expectations, gender inequity and biases persist in the United States workforce, resulting in unequal pay and discriminatory treatment [52, 53]. Women experience discrimination due to medical, domestic, and financial circumstances that position them at a disadvantage when competing with men during the hiring process and after they enter the workforce [48, 54–59]. Federal and state laws and policies have been enacted throughout the United States to address gender inequity and discrimination. In 1963, the United States implemented the Equal Pay Act, an amendment to the Fair Labor Standards Act, which protects all Americans against wage discrimination based on sex, regardless of region (Pub. L. No. 88–38, 77 Stat. 56 (1963)). However, even with a federally administered policy for wage equality, there is evidence for a persistent wage gap between men and women in the workforce [45, 55, 60], and the size of this gap can vary by region or state [55]. In addition to federal protections, several states have laws which require employers to provide equal pay for equal work (e.g., Massachusetts Equal Pay Act (Comm. Mass. § 149(105A) 2018), California Fair Pay Act (Cali. Labor Code § 432.3 & 1197.5 2017), Oregon Equal Pay Act (Ore. Stat. § 652(2110–235) 2019), Washington Equal Pay and Opportunities Act (Wash. Stat. § 49(58) 2019)). Other policies that promote equity for women in the workforce include paid family leave time, with some states offering up to 12 weeks of paid leave to eligible employees (e.g., CA, CT, MA, NJ, NY, OR, WA); although not exclusively beneficial for women, as women tend to take on a larger role in familial obligations, such policies tend to positively influence women’s career choices more than men’s [21–23, 25]. It is notable that many of these state-level policies that positively affect women are in states within the Pacific and East North Central regions, areas with greater gender parity in STEM tenure track jobs [47]. The spread of such policies (e.g., SD just instated a 12-week paid family leave policy, which will benefit faculty at the six state universities) will further encourage women in the workforce across the U.S. Additionally, national funding institutions are offering support for women in STEM, which would be available throughout the country. For instance, the National Science Foundation (NSF) offers grants through the ADVANCE program that aids in the retention and support of women; awards through this program are meant to develop mechanisms to alleviate systemic pressure women may experience within academia through research and policy. The NIH National Research Service Award offers flexible and family-centered initiatives that universities can promote to mothers to aid in parental care without needing to use their university funding to pay for leave; NSF also offers Career-Life Balance supplemental funding for existing awards to support research while senior researchers are on family leave. Utilizing such initiatives can help support women who may otherwise leave academia due to parental duties and insufficient funding to support them [61], however it is imperative that universities implement policies to aid in retention and inclusion, while also promoting applications for external resources.

In addition to policy differences, it is possible that women in academia may be less willing or unable to relocate following graduation as a result of partner restraints, family obligations, or other perceived cultural responsibilities [62, 63]; this could be especially true as many women in STEM graduate with their PhDs in their late 20s, a time when familial obligations also tend to increase. If women are less likely to move for a job, areas with higher proportions of women in STEM tenure track positions might be expected to overlap with areas graduating a higher proportion of women obtaining STEM PhDs. Although the states with the highest proportion of women with STEM PhDs (i.e., RI, MA, DE, VT, and MD all have higher than 45% women receiving PhDs in STEM fields [64]) do not necessarily have the highest number of women in tenure track positions, it is notable that the states with the lowest proportion of women graduating with STEM PhDs (i.e., SD (15.8%), WY (17.4%), MT (18.5%), ND (20.7%), and ID (22.1%) [64]) are all within areas we found here that had the fewest women in tenure track STEM positions.

Policies and programs must be in place to ensure equity in pay, hiring, and access to jobs for women in STEM, but mentorship and support are also important and must be available at every step, starting with recruitment of girls into the fields in K-12 and continuing into early careers to retain women. If support is offered to undergraduate women in STEM, they are more likely to perform well in introductory STEM courses [65, 66], which allows them to continue with their studies. Further, women trainees who receive mentorship from other women are more likely to remain in academia and have higher job satisfaction [31]. Fewer women scientist role models can also influence young women interested in science, causing them to feel misplaced and leading to inadequate mentoring, further contributing to attrition of women [31]. Decreased representation of women in K-12 or undergraduate STEM programs thus leads to issues in hiring and retention of women in graduate and faculty positions, creating a negative feedback cycle [9, 31, 67]. In addition, women are more likely to be hired in departments where women are already represented [9]. Groups that provide support for young girls and women in STEM, such as the National Girls Collaborative Project and the Association for Women in Science, as well as state run organizations, may help to provide mentorship, community, and financial support for the recruitment and retention of girls and women in STEM fields. Universities in regions with greater gender disparity might utilize tools such as those enacted by National Science Foundation through their ADVANCE program, which has been utilized by universities across the U.S. to improve the hiring and retention of women in STEM [16].

Although the use of perceived gender from online university profiles may not be reflective of an individual’s self-identified gender, understanding how departments are perceived by prospective colleagues and future generations of scientists has merit [26, 27]. We expect that non-binary or other marginalized students face additional obstacles in academia and having a role model of someone like them in the field is invaluable [21, 50, 68]. To create a more inclusive atmosphere, generally, and to promote more perspectives in the STEM fields, we encourage departments to consider listing pronouns on university websites. Without the inclusion of pronouns, students may not be able to determine how they would fit into a department and, overall, incorporating pronouns expresses a more inclusive and welcoming environment to any viewer.

There are many contributing factors to the attrition and slow advancement of women within academia in STEM, such as cultural and implicit biases, decreased grant reception, and imposter syndrome (feelings of self-doubt despite demonstrated accomplishments) [21, 62, 69–71]. Although the lack of women in academia is often blamed on the “leaky pipeline”, this should not be used as an excuse for inequities; women exist in the STEM fields at lower levels and should be better fostered to remain in academic STEM fields. Academic institutions should work towards solving these inequities by initiating plans to actively retain women faculty, such as alternative tenure clocks, childcare options, and paid leave policies. In this study, we chose to specifically focus on women and those perceived as women and the impact that STEM field and geographic region could have on gender equity in academia, due to the persistence of this well-known disparity. However, the same study could be done for other demographics (e.g., race) to understand how other marginalized communities may be viewing STEM fields. We show regional and departmental differences in gender disparity across the United States, to call attention to universities, academic fields, and particular regions that should focus on creating equal and inclusive opportunities for all underrepresented groups.

## Supporting information

S1 Table Classifications of selected institutions by location, region, size, and research activity level. (DOCX)

S2 Table Mean number and associated proportion of women and mean number of men employed in tenure track and instructor positions for each STEM department and division of the U.S. with the associated 95% credible interval. (DOCX)
