# Supplementary material for: Comparative analysis of gender disparity in academic positions based on U.S. region and STEM discipline
Source: PLoS One. 2024 Mar 20;19(3):e0298736. doi: 10.1371/journal.pone.0298736 (PMC10954166; doi:10.1371/journal.pone.0298736)
Supplement: S1 Table — (DOCX) [file pone.0298736.s001.docx]

**S1 Table**. Classifications of selected institutions by location, region, size, and research activity.

| Institution | Location | Region | Size | Research Activity Classification |
| --- | --- | --- | --- | --- |
| Adams State College | Alamosa, CO | Mountain | Medium | PUI |
| Adelphi University | Garden City, NY | Middle Atlantic | Medium | R2 |
| Alaska Pacific University | Anchorage, AK | Pacific | Small | PUI |
| Augustana College | Sioux Falls, SD | West North Central | Medium | PUI |
| Ball State University | Muncie, IN | East North Central | Large | R2 |
| Baylor University | Waco, TX | West South Central | Large | R1 |
| Bemidji State University | Bemidji, MN | West North Central | Medium | PUI |
| Bluefield State College | Bluefield, WV | South Atlantic | Medium | PUI |
| Boise State University | Boise, ID | Mountain | Large | R2 |
| Brigham Young University | Provo, UT | Mountain | Large | PUI |
| Brown University | Providence, RI | New England | Medium | R1 |
| California State University, Fresno | Fresno, CA | Pacific | Large | R2 |
| Carroll College | Helena, MT | Mountain | Medium | PUI |
| Carthage College | Kenosha, WI | East North Central | Medium | PUI |
| Chapman University | Orange, CA | Pacific | Medium | R2 |
| Clark University | Worcester, MA | New England | Medium | R2 |
| Clemson University | Clemson, SC | South Atlantic | Large | R1 |
| Coastal Carolina University | Conway, SC | South Atlantic | Large | PUI |
| Colby College | Waterville, ME | New England | Small | PUI |
| Colorado State University | Fort Collins, CO | Mountain | Large | R1 |
| Columbia College | Columbia, MO | West North Central | Large | PUI |
| Cornell University | Ithaca, NY | Middle Atlantic | Large | R1 |
| Creighton University | Omaha, NE | West North Central | Medium | R2 |
| Dartmouth College | Hanover, NH | New England | Medium | R1 |
| DePaul University | Chicago, IL | East North Central | Large | R2 |
| Dickinson College | Carlisle, PA | Middle Atlantic | Medium | PUI |
| Dixie State University | St. George, UT | Mountain | Medium | PUI |
| Duquesne | Pittsburgh, PA | Middle Atlantic | Medium | R2 |
| East Carolina University | Greenville, NC | South Atlantic | Large | R2 |
| East Tennessee State University | Johnson City, TN | East South Central | Large | R2 |
| Eastern Oregon University | La Grande, OR | Pacific | Medium | PUI |
| Fairfield University | Fairfield, CT | New England | Medium | PUI |
| Fordham University | Bronx, NY | Middle Atlantic | Large | R2 |
| Franklin College | Franklin, IN | East North Central | Medium | PUI |
| Frostburg State University | Frostburg, MD | South Atlantic | Medium | PUI |
| Furman University | Greenville County, SC | South Atlantic | Small | PUI |
| Georgetown College | Georgetown, KY | East South Central | Medium | PUI |
| Gonzaga University | Spokane, WA | Pacific | Medium | R2 |
| Hampton University | Hampton, VA | South Atlantic | Medium | PUI |
| Hawaii Pacific University | Honolulu, HI | Pacific | Medium | PUI |
| Huntington University | Huntington, IN | East North Central | Medium | PUI |
| Idaho State University | Pocatello, ID | Mountain | Large | R2 |
| Iowa State University | Ames, IA | West North Central | Large | R1 |
| Jackson State University | Jackson, MS | East South Central | Medium | R2 |
| Jamestown College | Jamestown, ND | West North Central | Small | PUI |
| Keene State College | Keene, NH | New England | Medium | PUI |
| La Salle University | Philadelphia, PA | Middle Atlantic | Medium | PUI |
| Lehigh University | Bethlehem, PA | Middle Atlantic | Medium | R2 |
| Lewis and Clark College | Portland, OR | Pacific | Small | R2 |
| Louisiana Tech University | Ruston, LA | West South Central | Large | R2 |
| Mercer University | Macon, GA | South Atlantic | Medium | R2 |
| Middle Tennessee State University | Murfreesboro, TN | East South Central | Large | R2 |
| Midland College | Midland, TX | West South Central | Medium | PUI |
| Missouri State University | Springfield, MO | West North Central | Large | PUI |
| Montana State University | Bozeman, MT | Mountain | Large | R1 |
| Montclair State University | Montclair, NJ | Middle Atlantic | Large | R2 |
| Nebraska Wesleyan University | Lincoln, NE | West North Central | Small | PUI |
| New Mexico Highlands University | Las Vegas, NM | Mountain | Small | PUI |
| North Dakota State University | Fargo, ND | West North Central | Large | R1 |
| Northern Arizona University | Flagstaff, AZ | Mountain | Large | R2 |
| Northern Michigan University | Marquette, MI | East North Central | Medium | R2 |
| Oakland University | Oakland County, MI | Midwest | Large | R2 |
| Oklahoma State University | Stillwater, OK | West South Central | Large | R1 |
| Old Dominion University | Norfolk, VA | South Atlantic | Large | R2 |
| Oregon State University | Corvallis, OR | Pacific | Large | R1 |
| Pacific University | Forest Grove, OR | Pacific | Small | PUI |
| Portland State University | Portland, OR | Pacific | Large | R2 |
| Purdue University | West Lafayette, IN | East North Central | Large | R1 |
| Rice University | Houston, TX | West South Central | Medium | R1 |
| Rider University | Lawrence Township, NJ | Middle Atlantic | Medium | PUI |
| Rowan University | Glassboro, NJ | Middle Atlantic | Large | R2 |
| Saint Louis University | St. Louis, MO | West North Central | Large | R2 |
| Sam Houston State University | Huntsville, TX | West South Central | Large | R2 |
| South Dakota School of Mines | Rapid City, SD | West North Central | Small | PUI |
| South Dakota State University | Brookings, SD | West North Central | Large | R2 |
| Southern University at New Orleans | New Orleans, LA | West South Central | Medium | PUI |
| Stanford University | Stanford, CA | Pacific | Large | R1 |
| Temple University | Philadelphia, PA | Middle Atlantic | Large | R1 |
| Tennessee State University | Nashville, TN | East South Central | Medium | R2 |
| Tennessee Technological University | Cookeville, TN | East South Central | Medium | R2 |
| Texas A&M University | Brazos County, TX | West South Central | Large | R1 |
| Texas Christian University | Forth Worth, TX | West South Central | Large | R2 |
| Trinity University | San Antonio, TX | West South Central | Medium | PUI |
| Union College | Schenectady, NY | Middle Atlantic | Small | PUI |
| Union University | Jackson, TN | East South Central | Medium | PUI |
| University of Alaska Anchorage | Anchorage, AK | Pacific | Large | R2 |
| University of New Orleans | New Orleans, LA | West South Central | Medium | R2 |
| University of Arkansas | Fayetteville, AR | West South Central | Large | R1 |
| University of Arkansas at Little Rock | Little Rock, AR | West South Central | Medium | R2 |
| University of California, Berkeley | Berkeley, CA | Pacific | Large | R1 |
| University of Cincinnati | Cincinnati, OH | East North Central | Large | R1 |
| University of Colorado Colorado Springs | Colorado Springs, CO | Mountain | Large | R1 |
| University of Illinois at Urbana Champaign | Champaign, IL | East North Central | Large | R1 |
| University of Louisville | Louisville, KY | East South Central | Large | R1 |
| University of Massachusetts Amherst | Hampshire County, MA | New England | Large | R1 |
| University of Massachusetts Lowell | Lowell, MA | New England | Large | R2 |
| University of Minnesota Twin Cities | Minneapolis, MN | West North Central | Large | R1 |
| University of Mississippi | Oxford, MS | East South Central | Large | R1 |
| University of Montana | Missoula, MT | Mountain | Large | R2 |
| University of Montevallo | Montevallo, AL | East South Central | Medium | PUI |
| University of Nebraska at Lincoln | Lincoln, NE | West North Central | Large | R1 |
| University of Nebraska at Omaha | Omaha, NE | West North Central | Large | R1 |
| University of New England | Biddeford, ME | New England | Large | R2 |
| University of New Mexico | Albuquerque, NM | Mountain | Large | R1 |
| University of North Florida | Jacksonville, FL | South Atlantic | Large | R2 |
| University of Portland | Portland, OR | Pacific | Medium | PUI |
| University of Rhode Island | South Kingstown, RI | New England | Large | R1 |
| University of Rochester | Monrow County, NY | Middle Atlantic | Large | R1 |
| University of San Diego | San Diego, CA | Pacific | Medium | R2 |
| University of South Alabama | Mobile, AL | East South Central | Medium | R2 |
| University of South Dakota | Vermillion, SD | West North Central | Large | R2 |
| University of South Florida | Hillsborough County, FL | South Atlantic | Large | R1 |
| University of Southern Mississippi | Hattiesburg, MS | East South Central | Large | R1 |
| University of Vermont | Chittenden County, VT | New England | Large | R2 |
| University of Washington | Seattle, WA | Pacific | Large | R1 |
| University of Wisconsin Stout | Menomonie, WI | East North Central | Large | R2 |
| University of Wyoming | Laramie, WY | Mountain | Large | R2 |
| Valparaiso University | Porter County, IN | East North Central | Medium | R2 |
| Wake Forest University | Winston-Salem, NC | South Atlantic | Medium | R2 |
| Washington State University | Pullman, WA | Pacific | Large | R1 |
| Wayne State University | Detroit, MI | East North Central | Large | R1 |
| West Virginia University | Morgantown, WV | South Atlantic | Large | R1 |
| Westminster College | Fulton, MO | Mountain | Small | PUI |
| Winona State University | Winona, MN | West North Central | Medium | PUI |
| Worcester Polytechnic Institute | Worcester, MA | New England | Medium | R2 |
| Worcester State University | Worcester, MA | New England | Medium | PUI |
| Yale University | New Haven, CT | New England | Large | R1 |
