# Supplementary material for: Comparative analysis of gender disparity in academic positions based on U.S. region and STEM discipline
Source: PLoS One. 2024 Mar 20;19(3):e0298736. doi: 10.1371/journal.pone.0298736 (PMC10954166; doi:10.1371/journal.pone.0298736)
Supplement: S2 Table — (DOCX) [file pone.0298736.s002.docx]

**S2 Table**. Mean number and associated proportion of women and mean number of men employed in tenure track and instructor positions for each STEM department and division of the US with the associated 95% credible interval.

| Division | Department | Position | Number of Women | Number of Men | Proportion of Women |
| --- | --- | --- | --- | --- | --- |
| East North Central | Biology | Instructor | 5.3 (4.4-6.4) | 4.7 (3.7-5.8) | 0.55 (0.48-0.61) |
| East North Central | Biology | Tenure track | 13.3 (11.4-15.3) | 24.8 (21.9-27.8) | 0.36 (0.32-0.40) |
| East North Central | Chemistry | Instructor | 3.4 (2.5-4.5) | 3.8 (2.7-5.1) | 0.47 (0.37-0.57) |
| East North Central | Chemistry | Tenure track | 6.0 (4.7-7.4) | 12.4 (10.3-14.6) | 0.32 (0.27-0.38) |
| East North Central | Computer science | Instructor | 2.5 (1.7-3.6) | 6.5 (4.5-8.8) | 0.29 (0.20-0.38) |
| East North Central | Computer science | Tenure track | 4.1 (3.0-5.5) | 18.3 (15.3-21.5) | 0.19 (0.14-0.44) |
| East North Central | Engineering | Instructor | 8.9 (6.9-11.2) | 25.2 (21.2-29.7) | 0.25 (0.20-0.30) |
| East North Central | Engineering | Tenure track | 57.2 (50.8-63.8) | 215.4 (202.6-228.4) | 0.21 (0.19-0.23) |
| East North Central | Mathematics | Instructor | 6.6 (5.1-8.3) | 5.7 (4.3-7.3) | 0.53 (0.45-0.61) |
| East North Central | Mathematics | Tenure track | 6.3 (5.0-7.8) | 20.6 (17.9-23.4) | 0.24 (0.20-0.29) |
| East North Central | Physics | Instructor | 1.8 (1.2-2.5) | 3.6 (2.6-5.2) | 0.30 (0.20-0.40) |
| East North Central | Physics | Tenure track | 4.2 (3.1-5.6) | 18.1 (15.3-21.1) | 0.19 (0.14-0.23) |
| East South Central | Biology | Instructor | 2.5 (1.6-3.7) | 1.5 (0.8-2.5) | 0.64 (0.47-0.74) |
| East South Central | Biology | Tenure track | 5.5 (4.0-7.1) | 11.7 (9.5-14.1) | 0.29 (0.23-0.36) |
| East South Central | Chemistry | Instructor | 3.1 (1.7-4.8) | 1.7 (0.8-3.1) | 0.62 (0.47-0.77) |
| East South Central | Chemistry | Tenure track | 3.8 (2.6-5.3) | 11.0 (8.8-13.4) | 0.24 (0.18-0.31) |
| East South Central | Computer science | Instructor | 2.2 (1.1-3.9) | 4.1 (2.3-6.6) | 0.37 (0.22-0.54) |
| East South Central | Computer science | Tenure track | 2.4 (1.4-3.6) | 8.6 (6.6-10.9) | 0.21 (0.13-0.29) |
| East South Central | Engineering | Instructor | 1.7 (0.9-2.8) | 3.5 (2.2-5.1) | 0.42 (0.30-0.55) |
| East South Central | Engineering | Tenure track | 5.8 (4.2-7.7) | 25.5 (21.9-29.4) | 0.17 (0.14-0.22) |
| East South Central | Mathematics | Instructor | 3.1 (1.8-4.6) | 2.4 (1.3-3.8) | 0.61 (0.49-0.73) |
| East South Central | Mathematics | Tenure track | 3.9 (2.7-5.3) | 10.6 (8.5-12.9) | 0.28 (0.22-0.35) |
| East South Central | Physics | Instructor | 1.0 (0.4-1.7) | 1.1 (0.5-2.0) | 0.48 (0.29-0.67) |
| East South Central | Physics | Tenure track | 1.2 (0.6-2.1) | 7.0 (5.3-9.0) | 0.14 (0.08-0.21) |
| Middle Atlantic | Biology | Instructor | 2.2 (1.6-2.9) | 1.7 (1.1-2.3) | 0.40 (0.31-0.48) |
| Middle Atlantic | Biology | Tenure track | 5.6 (4.6-6.8) | 11.6 (10.0-13.2) | 0.14 (0.12-0.17) |
| Middle Atlantic | Chemistry | Instructor | 1.8 (1.0-2.9) | 2.7 (1.6-4.1) | 0.37 (0.22-0.52) |
| Middle Atlantic | Chemistry | Tenure track | 4.3 (3.2-5.6) | 10.8 (9.0-12.8) | 0.29 (0.22-0.35) |
| Middle Atlantic | Computer science | Instructor | 2.7 (1.6-4.0) | 5.6 (3.9-7.7) | 0.30 (0.20-0.42) |
| Middle Atlantic | Computer science | Tenure track | 3.9 (2.7-5.3) | 16.1 (13.4-19.0) | 0.18 (0.13-0.24) |
| Middle Atlantic | Engineering | Instructor | 3.7 (2.4-4.7) | 7.8 (5.3-10.6) | 0.30 (0.20-0.42) |
| Middle Atlantic | Engineering | Tenure track | 16.5 (13.1-20.1) | 51.2 (45.1-57.7) | 0.25 (0.21-0.29) |
| Middle Atlantic | Mathematics | Instructor | 3.5 (2.4-4.7) | 3.0 (2.0-4.1) | 0.54 (0.43-0.65) |
| Middle Atlantic | Mathematics | Tenure track | 3.8 (2.7-5.1) | 14.1 (11.9-16.5) | 0.20 (0.15-0.26) |
| Middle Atlantic | Physics | Instructor | 0.8 (0.4-1.5) | 1.4 (0.6-2.4) | 0.37 (0.18-0.58) |
| Middle Atlantic | Physics | Tenure track | 2.6 (1.7-3.7) | 11.8 (9.7-14.2) | 0.15 (0.11-0.21) |
| Mountain | Biology | Instructor | 2.8 (1.9-3.9) | 1.7 (1.0-2.6) | 0.60 (0.51-0.70) |
| Mountain | Biology | Tenure track | 5.3 (4.0-6.7) | 12.2 (10.1-14.5) | 0.24 (0.21-0.28) |
| Mountain | Chemistry | Instructor | 3.5 (2.2-5.2) | 3.1 (1.8-4.8) | 0.54 (0.43-0.65) |
| Mountain | Chemistry | Tenure track | 4.7 (3.4-6.3) | 12.0 (9.6-14.6) | 0.26 (0.21-0.32) |
| Mountain | Computer science | Instructor | 1.1 (0.4-2.2) | 4.0 (2.1-6.4) | 0.30 (0.18-0.44) |
| Mountain | Computer science | Tenure track | 1.6 (0.8-2.7) | 14.5 (11.6-17.7) | 0.17 (0.12-0.23) |
| Mountain | Engineering | Instructor | 3.3 (2.0-4.9) | 8.5 (6.1-11.2) | 0.18 (0.12-0.25) |
| Mountain | Engineering | Tenure track | 9.3 (7.2-11.7) | 41.3 (36.7-46.2) | 0.11 (0.09-0.13) |
| Mountain | Mathematics | Instructor | 6.9 (4.9-9.3) | 4.9 (3.2-7.0) | 0.55 (0.46-0.63) |
| Mountain | Mathematics | Tenure track | 4.4 (3.2-5.6) | 14.3 (11.9-16.9) | 0.21 (0.17-0.26) |
| Mountain | Physics | Instructor | 1.0 (0.4-1.7) | 1.5 (0.8-2.6) | 0.37 (0.22-0.53) |
| Mountain | Physics | Tenure track | 1.5 (0.8-2.5) | 10.5 (8.2-13.0) | 0.15 (0.10-0.20) |
| New England | Biology | Instructor | 2.8 (2.0-3.7) | 1.6 (1.1-2.3) | 0.48 (0.40-0.56) |
| New England | Biology | Tenure track | 10.6 (8.9-12.4) | 15.0 (13.0-17.3) | 0.17 (0.15-0.19) |
| New England | Chemistry | Instructor | 1.9 (1.2-2.9) | 2.5 (1.6-3.7) | 0.42 (0.31-0.54) |
| New England | Chemistry | Tenure track | 3.9 (2.7-5.4) | 10.4 (8.3-12.8) | 0.27 (0.21-0.34) |
| New England | Computer science | Instructor | 2.4 (1.4-3.8) | 6.4 (4.5-8.8) | 0.32 (0.22-0.43) |
| New England | Computer science | Tenure track | 5.1 (3.6-6.9) | 18.1 (15.1-21.4) | 0.21 (0.16-0.27) |
| New England | Engineering | Instructor | 2.9 (1.7-4.4) | 7.7 (5.5-10.2) | 0.26 (0.19-0.34) |
| New England | Engineering | Tenure track | 19.7 (16.0-23.7) | 71.3 (64.1-78.9) | 0.21 (0.18-0.24) |
| New England | Mathematics | Instructor | 2.8 (1.9-3.9) | 3.3 (2.3-4.6) | 0.44 (0.35-0.53) |
| New England | Mathematics | Tenure track | 4.1 (2.9-5.4) | 14.9 (12.6-17.4) | 0.15 (0.12-0.19) |
| New England | Physics | Instructor | 1.6 (0.9-2.7) | 2.1 (1.1-3.5) | 0.34 (0.21-0.49) |
| New England | Physics | Tenure track | 3.2 (2.0-4.5) | 16.1 (13.3-19.2) | 0.17 (0.12-0.23) |
| Pacific | Biology | Instructor | 5.5 (4.2-7.0) | 3.0 (2.0-4.1) | 0.64 (0.56-0.71) |
| Pacific | Biology | Tenure track | 9.0 (7.4-10.7) | 11.7 (9.9-13.7) | 0.41 (0.36-0.46) |
| Pacific | Chemistry | Instructor | 4.5 (3.3-5.9) | 4.6 (3.3-6.0) | 0.49 (0.40-0.57) |
| Pacific | Chemistry | Tenure track | 3.7 (2.7-4.9) | 10.2 (8.5-12.1) | 0.25 (0.20-0.30) |
| Pacific | Computer science | Instructor | 2.1 (1.3-3.2) | 3.3 (2.1-4.7) | 0.35 (0.23-0.48) |
| Pacific | Computer science | Tenure track | 5.3 (3.8-7.0) | 18.7 (15.8-21.8) | 0.23 (0.18-0.28) |
| Pacific | Engineering | Instructor | 6.8 (4.9-9.0) | 12.4 (9.7-15.3) | 0.30 (0.24-0.36) |
| Pacific | Engineering | Tenure track | 19.4 (16.5-22.7) | 66.8 (61.3-72.5) | 0.22 (0.20-0.25) |
| Pacific | Mathematics | Instructor | 6.7 (5.0-8.7) | 7.4 (5.6-9.5) | 0.46 (0.39-0.54) |
| Pacific | Mathematics | Tenure track | 3.9 (2.9-5.0) | 14.1 (12.2-16.2) | 0.20 (0.16-0.24) |
| Pacific | Physics | Instructor | 1.2 (0.6-1.9) | 3.1 (2.1-4.3) | 0.22 (0.14-0.32) |
| Pacific | Physics | Tenure track | 3.7 (2.6-4.9) | 13.7 (11.5-16.1) | 0.16 (0.12-0.20) |
| South Atlantic | Biology | Instructor | 5.3 (4.0-6.8) | 3.7 (2.6-5.1) | 0.51 (0.44-0.59) |
| South Atlantic | Biology | Tenure track | 7.1 (5.6-8.8) | 11.1 (9.0-13.3) | 0.33 (0.28-0.38) |
| South Atlantic | Chemistry | Instructor | 4.2 (2.8-5.7) | 4.1 (2.7-5.6) | 0.47 (0.37-0.58) |
| South Atlantic | Chemistry | Tenure track | 3.1 (2.0-4.4) | 9.1 (7.1-11.3) | 0.25 (0.19-0.32) |
| South Atlantic | Computer science | Instructor | 2.6 (1.7-3.8) | 3.6 (2.4-5.0) | 0.36 (0.0.26-0.48) |
| South Atlantic | Computer science | Tenure track | 3.5 (2.3-4.8) | 9.4 (7.4-11.7) | 0.25 (0.19-0.31) |
| South Atlantic | Engineering | Instructor | 1.1 (0.7-1.7) | 2.1 (1.4-3.0) | 0.09 (0.06-0.13) |
| South Atlantic | Engineering | Tenure track | 3.6 (2.7-4.7) | 15.3 (13.4-17.5) | 0.05 (0.04-0.06) |
| South Atlantic | Mathematics | Instructor | 7.3 (5.6-9.2) | 5.4 (4.0-7.1) | 0.56 (0.48-0.64) |
| South Atlantic | Mathematics | Tenure track | 3.3 (2.2-4.6) | 13.3 (10.9-15.9) | 0.23 (0.18-0.28) |
| South Atlantic | Physics | Instructor | 1.1 (0.6-1.9) | 3.3 (2.1-4.7) | 0.22 (0.12-0.34) |
| South Atlantic | Physics | Tenure track | 1.5 (0.8-2.4) | 11.9 (9.5-14.5) | 0.14 (0.10-0.20) |
| West North Central | Biology | Instructor | 4.5 (3.2-6.0) | 2.5 (1.6-3.6) | 0.67 (0.58-0.76) |
| West North Central | Biology | Tenure track | 12.6 (10.5-14.8) | 20.8 (18.1-23.7) | 0.37 (0.32-0.41) |
| West North Central | Chemistry | Instructor | 2.6 (1.5-3.9) | 2.8 (1.7-4.2) | 0.48 (0.36-0.61) |
| West North Central | Chemistry | Tenure track | 3.8 (2.6-5.1) | 10.2 (8.2-12.4) | 0.25 (0.20-0.31) |
| West North Central | Computer science | Instructor | 2.2 (1.0-4.1) | 4.6 (2.3-7.8) | 0.32 (0.19-0.47) |
| West North Central | Computer science | Tenure track | 2.5 (1.4-3.8) | 7.5 (5.5-9.8) | 0.23 (0.16-0.30) |
| West North Central | Engineering | Instructor | 6.0 (4.0-8.5) | 18.1 (14.2-22.4) | 0.25 (0.19-0.33) |
| West North Central | Engineering | Tenure track | 16.2 (12.5-20.3) | 76.9 (68.8-85.6) | 0.18 (0.15-0.21) |
| West North Central | Mathematics | Instructor | 3.8 (2.4-5.5) | 3.8 (2.3-5.6) | 0.52 (0.43-0.61) |
| West North Central | Mathematics | Tenure track | 2.8 (1.8-4.0) | 10.0 (7.9-12.2) | 0.22 (0.18-0.27) |
| West North Central | Physics | Instructor | 1.2 (0.5-2.2) | 2.0 (0.9-3.5) | 0.28 (0.15-0.44) |
| West North Central | Physics | Tenure track | 1.6 (0.9-2.5) | 8.6 (6.6-10.7) | 0.14 (0.10-0.19) |
| West South Central | Biology | Instructor | 5.0 (3.8-6.2) | 3.8 (2.8-5.0) | 0.57 (0.49-0.66) |
| West South Central | Biology | Tenure track | 5.9 (4.8-7.3) | 13.3 (11.4-15.3) | 0.30 (0.25-0.36) |
| West South Central | Chemistry | Instructor | 4.0 (2.9-5.2) | 2.8 (1.9-3.9) | 0.59 (0.47-0.69) |
| West South Central | Chemistry | Tenure track | 3.0 (2.1-4.0) | 11.9 (10.1-13.9) | 0.20 (0.15-0.26) |
| West South Central | Computer science | Instructor | 1.9 (1.1-2.9) | 5.9 (4.3-7.8) | 0.24 (0.14-0.35) |
| West South Central | Computer science | Tenure track | 2.3 (1.5-3.2) | 11.4 (9.6-13.4) | 0.17 (0.12-0.23) |
| West South Central | Engineering | Instructor | 9.5 (7.5-11.7) | 29.4 (25.8-33.2) | 0.24 (0.20-0.29) |
| West South Central | Engineering | Tenure track | 15.8 (13.3-18.5) | 81.3 (75.6-87.2) | 0.16 (0.14-0.19) |
| West South Central | Mathematics | Instructor | 5.8 (4.5-7.2) | 6.2 (4.8-7.8) | 0.48 (0.40-0.56) |
| West South Central | Mathematics | Tenure track | 3.5 (2.6-4.6) | 15.6 (13.5-17.8) | 0.19 (0.14-0.23) |
| West South Central | Physics | Instructor | 1.0 (0.5-1.6) | 2.2 (1.4-3.2) | 0.28 (0.15-0.44) |
| West South Central | Physics | Tenure track | 2.5 (1.7-3.4) | 12.9 (11.0-14.9) | 0.16 (0.12-0.22) |
